# Network analysis of additional clinical features of (Internet) gaming disorder

**DOI:** 10.1002/mpr.2021

**Published:** 2024-05-27

**Authors:** Marcel Martončik, Matúš Adamkovič, Ivan Ropovik

**Affiliations:** ^1^ Institute of Social Sciences CSPS SAS Košice Slovakia; ^2^ Faculty of Humanities and Social Sciences University of Jyväskylä Jyväskylä Finland; ^3^ Faculty of Education Charles University Prague Czechia; ^4^ Faculty of Education University of Presov Prešov Slovakia; ^5^ Institute of Psychology Czech Academy of Sciences Prague Czechia

**Keywords:** diagnostic, functional impairment, gaming disorder, network analysis, network approach

## Abstract

**Objectives:**

There are dozens of screening instruments purporting to measure the (Internet) gaming disorder (IGD/GD). The two prominent diagnostic manuals, DSM‐5 and ICD‐11, list several additional diagnostic or clinical features and problems (e.g., neglect of sleep, neglect of daily duties, health deterioration) that should co‐occur or be caused by the IGD/GD. It remains unclear how specific IGD/GD operationalizations (different screening scales) are related to these functional impairments.

**Methods:**

To explore this, data on six measures of IGD/GD (IGDS9‐SF, GDSS, GDT, GAMES test, two self‐assessments) and 18 additional diagnostic features were collected from a sample of 1009 players who play digital games at least 13 h per week. A network approach was utilized to determine which operationalization is most strongly associated with functional impairment.

**Results:**

In most of the networks, IGD/GD consistently emerged as the most central node.

**Conclusion:**

The similar centrality of IGD/GD, irrespective of its definition (DSM‐5 or ICD‐11) or operationalization, provides support for the valid comparison or synthesis of results from studies that used instruments coming from both DSM‐5 and ICD‐11 ontologies, but only if the goal is to evaluate IGD/GD relationships to other phenomena, not the relationships between the symptoms themselves.

## 1 INTRODUCTION

In January 2022, the 11th Revision of the International Classification of Diseases (ICD) came into effect (WHO, 2019) and officially recognized gaming disorder (GD) as its second behavioral addiction disorder. Gaming disorder is defined as “a pattern of persistent or recurrent gaming behavior” manifested by three symptoms: (1) impaired control over gaming, (2) increasing priority given to gaming, and (3) continuation of gaming despite the occurrence of negative consequences. These symptoms were believed to represent the core of the previously suggested nine symptoms^1^ of Internet gaming disorder, (IGD) (APA, 2013), a predecessing construct suggested in DSM‐5 as a condition for further research. It is estimated from representative studies that around 2.4% (Kim et al., 2022) of people worldwide may show these symptoms and suffer from GD. A recent meta‐analysis (Ropovik et al., 2022) linked several variables as potential risk factors of GD including escape motivation, depression, Internet or social networking addiction, stress, gaming time, impulsivity, and anxiety.

Recently, some researchers (e.g., Király et al., 2022; Reed et al., 2022) had expected that the adoption of ICD‐11 would provide validity (i.e., by defining core symptoms) and bring more unity (i.e., utilizing fewer, unified measurement tools) to the conceptualization and measurement of GD, especially in comparison to DSM‐5's IGD. The DSM‐5's description of IGD have resulted in the development of various screening scales with inconsistencies in symptom selection and operationalization (Karhulahti et al., 2021; King, Chamberlain, et al., 2020). However, the usage of screening measures based on different ontologies (including DSM‐5 and ICD‐11) may identify different groups of individuals (Ko et al., 2020a; Starcevic et al., 2020) with distinct psychological and health characteristics (Karhulahti et al., 2022). Moreover, within the same ontological definition, diverse ways of operationalizing the same symptoms can substantially affect the relationships between the symptoms (Adamkovič et al., 2023).

On the other hand, the three “essential features” (symptoms) of GD (WHO, 2019) were initially proposed to help distinguish between pathological and non‐pathological gaming. In one of the updates of ICD‐11 (1/2023) several “additional features” of GD have been added, for instance: increased duration or frequency of gaming, craving, or exhibition of adversarial behavior or aggression. It is yet to be seen whether the addition of the new features, given the absence of unified measures, will lead to the development of multiple new screening scales (GDT, Pontes et al., 2019; GADIS‐A, Paschke et al., 2020; GDHGS, Balhara et al., 2020; GAMES test, Higuchi et al., 2021; LMGDQ, Lee et al., 2022; GDSS, Lyu et al., 2022; GDSQ, Zhang et al., 2022) as we have seen with the DSM‐5 (King, Billieux, et al., 2020).

Both the DSM‐5 and ICD‐11 manuals thus outline several problems or negative consequences typically associated with IGD/GD. Combined, they include neglect of eating, sleeping, normal obligations, or interpersonal activities, avoidance of boredom, time spent gaming, aggression, declining grades, health and sleep problems. Solid evidence has been gathered for many other negative outcomes such as loneliness, anxiety, depression, emotional distress, lower life satisfaction, or poor self‐esteem (Richard et al., 2020; Ropovik et al., 2022).

### 1.1 Network approach

The network approach to psychopathology (e.g., Borsboom, 2017; Borsboom & Cramer, 2013) transitions the traditional perspective of viewing constructs as latent entities toward conceptualizing them as complex systems. These systems emerge from mutual and recurrent interactions of causal agents, typically symptoms. The network approach thus allows to study and communicate the complexity of the relationships within the entire network. The network analysis is a tool that helps identify the structure and dynamics of a network, including the estimation of the centrality and connectivity of agents within the network. The relationships between the agents (nodes) can also be visualized—a connection between two nodes indicate that the nodes are conditionally dependent whereas no connection between two nodes indicate that the nodes are conditionally independent. The more connections a node has, and the stronger these connections are, the greater its centrality within the network. Although the network approach in psychopathology is typically applied to a single concept or comorbidity (e.g., Robinaugh et al., 2020), it can be highly informative in identifying core elements within a network comprising several distinct variables.

### 1.2 Present study

In the present exploratory study, *we aim to examine which IGD/GD operationalization is most strongly related to the additional diagnostic/clinical features or problems typically associated with IGD/GD as a functional impairment*. Following the network approach to psychopathology, we perceive these additional diagnostic features to form a complex network of relationships. Within this network, IGD or GD, by definition, should play the most central role. The available evidence, however, suggests that different symptoms and ontological definitions of GD in general have varying degrees of usefulness and diagnostic utility. For instance, an international Delphi study conducted by Castro‐Calvo et al. (2021) reported insufficient or low diagnostic validity of some IGD criteria, while GD criteria were deemed to have high diagnostic validity and clinical utility (Billieux et al., 2019). Understanding the structural interconnectedness between different combinations of IGD/GD operationalizations and the related problems could be important for the recent WHO Collaborative Project on the Development of New International Screening and Diagnostic Instruments for GD (Carragher et al., 2022), which aims to distinguish pathological from non‐pathological gaming, taking into account differences between DSM‐5 and ICD‐11.

## 2 METHOD

Ethical approval to conduct the study was granted by the Ethics committee of the Faculty of Arts, University of Presov.

### 2.1 Participants

Participants (*N* = 1009) were recruited via the Prolific platform. The sample comprised players who play digital games on any device for at least 13 h per week and considered gaming to be their favorite hobby (defined as two pre‐specified inclusion criteria on Prolific). The average age of the participants was 29.80 years old (*SD* = 9.47), with 797 males, 191 females, and 21 non‐binary people. Their daily gaming time was on average 4.57 h (*SD* = 2.85). About half of the participants were residents of the UK and the US, and the rest were mostly from Europe.

Disordered gaming was treated as a continuous variable, spanning from non‐pathological to extremely pathological, instead of a taxon (see Haslam et al., 2020). To prevent introducing selection bias (de Ron et al., 2021), data of the entire sample was analyzed. Subsetting only players above a certain cut‐off on a symptom sum score could lead to downward bias and possibly spurious, non‐causal edges. This is because IGD/GD sum score is by definition a common effect of pairs of nodes in the network. Assuming that the symptomatology of IGD/GD defined in the respective diagnostic manuals includes all clinically relevant symptoms, partial correlations between the network nodes are expected to be unbiased only if sample selection is not conditioning on a common effect of those nodes.

### 2.2 Measures

To measure IGD, we employed the IGDS9‐SF, which is currently the most frequently cited instrument grounded in DSM‐5 ontology (9 items; Pontes & Griffiths, 2015; *ω*_total_ = 0.92).

For ICD‐11‐based GD, we used the Gaming Disorder Test (4 items; Pontes et al., 2019; *ω*_total_ = 0.90), which is the most‐cited measure and has the highest content validity (see Karhulahti et al., 2021). In addition, we utilized two recently developed instruments that operationalize GD in a different way—incorporating also the DSM‐5 definition of IGD, evidence pertaining to cognitive factors underlying IGD, and feedback from community sample groups and treatment seekers. These are the Gaming Disorder Screening Scale—GDSS (18 items; Lyu et al., 2022; *ω*_total_ = 0.94), and the GAMing Engagement Screener test—GAMES test (9 items; Higuchi et al., 2021; *ω*_total_ = 0.86).

A single‐item THL1 (Salonen & Raisamo, 2015) was used to self‐assess gaming problems. Another self‐assessment of the pathological pattern of gaming was operationalized using the following five‐point Likert scale item: “Do you think that playing digital games over the last 12 months caused you such problems that would make you seek psychological or psychiatric help?”.

We further measured nine additional diagnostic features reported in the DSM‐5 (APA, 2013; Diagnostic features, p. 796–797), specifically: (1) gaming time, (2) neglect of food, (3) neglect of sleep, (4) neglect of daily duties or responsibilities, (5) boredom avoidance motivation, (6) preference for multiplayer games, (7) preference for competitive play (esports), and (8) neglect of interpersonal activities. In addition, one functional correlate reported in the DSM‐5—decline in school grades—was measured.

Five additional clinical features reported in the ICD‐11 were administered, specifically: (1) craving, (2) aggression upon cessation of gaming, (3) disruptions in dietary habits, (4) health deterioration, and (5) negative mental health outcome measured as well‐being (Warwick‐Edinburgh Mental Well‐being Scale—WEMWBS; Tennant et al., 2007, *ω*_total_ = 0.89; see also ICD‐11). When selecting additional clinical features, we aimed to avoid making selective choices and instead include all that were already identified. The only exception concerns comorbidity, encompassing six constructs, due to the feasibility of data collection. The remaining two comorbid disorders (Attention Deficit Hyperactivity Disorder and Obsessive‐Compulsive Disorder) listed in the ICD 11 (WHO, 2019) were not included. Detailed information about the operationalizations of all clinical/diagnostic features is available at: https://osf.io/x5f2e.

Comorbidity (as a part of Additional clinical features in ICD‐11) in the form of substance abuse was measured using a single item “How often has it happened over the last 12 months that you have used the following substances: alcohol, tobacco products, soft drugs, or hard drugs?” on a 5 point ordinal scale. Depression and anxiety was measured using the Patient Health Questionnaire for Depression and Anxiety—PHQ‐4 (4 items; Kroenke et al., 2009; *ω*_total_ = 0.92 and 0.87 for anxiety and depression, respectively). Based on the review by Richard et al. (2020) we also measured loneliness (a potential consequence of neglect of interpersonal activities and family, reported in the DSM‐5) and self‐esteem (mentioned in the ICD‐11 as a frequent correlate). Loneliness was measured using the De Jong Gierveld Loneliness Scale (6 items; De Jong Gierveld and Van Tilburg (2006); *ω*_total_ = 0.87). Global self‐esteem was measured using the single item reported in Robins et al. (2001). All scales were administered in a randomized order to mitigate the order effects.

### 2.3 Statistical analysis

The data were first screened for improbable responses and careless responding patterns. We excluded participants who were either (1) being identified as multivariate outliers using the Mahalanobis distance or (2) failing two out of three attention checks. In total, 8.4% of the participants were dropped. For each scale that had three or more items, a confirmatory factor model was estimated and the respective factor score was extracted. For the two‐item measures, PCA was computed, and the component score was extracted.

To gain insight into the interrelationships between the variables, six network models were estimated. Each network model included a single IGD/GD operationalization and 20 additional diagnostic features or negative outcomes as mentioned above. The networks were estimated using the EBICglasso method, inputting polychoric correlations, with the tuning parameter set to.25^2^. Given the goals of the study, we focused our interpretations specifically on the strength centrality parameter (i.e., the sum of a node's connections' absolute weights). The emphasis on strength stems from its ability to convey the magnitude of a node's connectedness, regardless of the sign (positive or negative) of these connections. To examine the accuracy and stability of the observed strength coefficients and edge‐weights, bootstrapping (2000 samples) was performed, showing sufficient stability of the estimates (see https://osf.io/qcr9u). To compare the strength coefficient of different IGD/GD operationalizations across the networks, the network comparison test with 1000 iterations was conducted. To compare the strength indices of the nodes within the networks, bootstrapped (2000 samples) difference tests were calculated. All the analyses were performed in R version 4.3.1 using lavaan (Rosseel, 2012), bootnet (Epskamp et al., 2018) and NetworkComparisonTest (van Borkulo et al., 2017) as the main packages. The data and R script are available on the OSF repository: https://osf.io/by6d2/.

## 3 RESULTS

Descriptive statistics are presented in Table 1.

**TABLE 1:** Descriptive statistics.

	*M*	*SD*	Potential range
GDT	2.00	0.89	1–5
IGDS9‐SF	1.99	0.72	1–5
GDSS	1.62	0.45	1–4
GAMES test	0.52	0.26	0–3
THL1	1.48	0.83	1–5
Self‐assessment	1.57	0.68	1–4
Gaming time	4.54	2.71	1–24
Disrupted diet	2.27	1.18	1–5
Missed food	2.09	1.11	1–5
Missed sleep	2.59	1.15	1–5
Neglect of duties	2.03	1.03	1–5
Multiplayer (percentage of time spent playing multiplayer)	43.27	32.86	0–100
Esports game (1 = no, 2 = yes)	1.78	0.42	1–2
Boredom avoidance	3.51	1.04	1–5
Offline contact (hours per day)	3.43	5.00	0–24
Other gaming activities (hours per day)	3.49	5.06	0–24
Craving	2.23	1.06	1–5
Aggression	1.29	0.66	1–5
Grades decline	1.62	0.98	1–6
Health deterioration	2.00	1.06	1–5
Usage of drugs	2.13	1.30	1–5
Anxiety	2.08	0.92	1–4
Depression	2.08	0.88	1–4
Loneliness	2.73	0.93	1–5
Self‐confidence	2.68	1.25	1–5
Wellbeing	3.28	0.76	1–5

**FIGURE 1:**
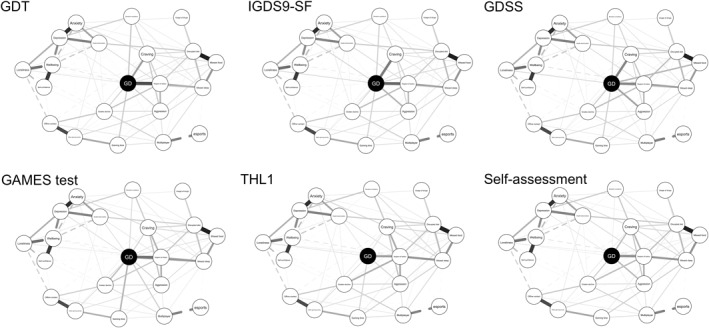
Visualization of the networks.

**FIGURE 2:**
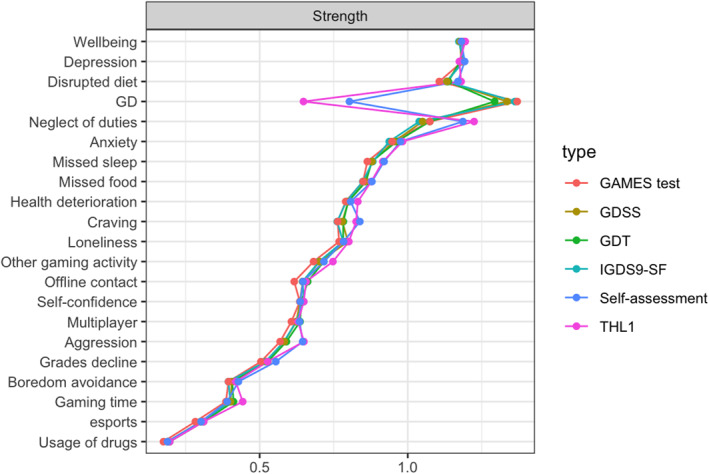
Nodes' strength parameter across different IGD/GD operationalizations.

**FIGURE 3:**
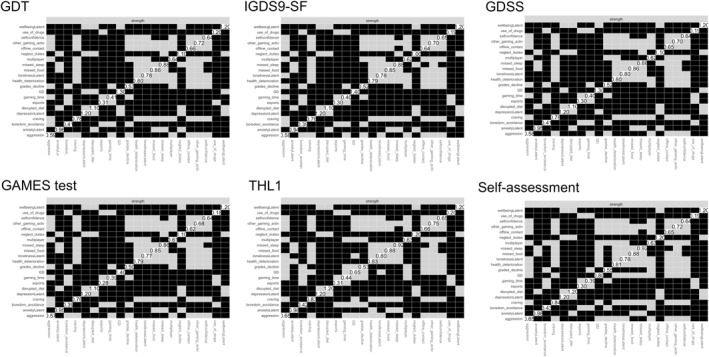
Bootstrapped difference tests for the nodes' strength within IGD/GD networks.

Network structures differing in the IGD/GD operationalization can be seen in Figure 1, with the GD node being displayed black. Values of the strength parameter for all nodes across different IGD/GD operationalizations are visualized in Figure 2. The highest value of strength (i.e., a node's overall dominance in the network) was observed for the ICD‐11‐based operationalization used by the GAMES test, followed by IGDS9‐SF (DSM‐5‐based), GDSS, and GDT (both ICD‐11‐based). However, the differences in strength among these four operationalizations, as estimated using the network comparison test, were non‐significant (*p‐values* ranging from 0.429 to 0.930). A significant drop in the strength of the GD node was observed for GD self‐assessment and the single‐item THL1 (*p‐values* < 0.001 compared to the other four operationalizations). The full results of the network comparison test are presented in Table 2. Summaries of the bootstrapped difference tests for the nodes' strength within each network are visualized in Figure 3.

**TABLE 2:** Results of network comparison tests for the strength parameter across different IGD/GD operationalizations.

	GDT	IGDS9‐SF	GDSS	GAMES test	THL1	Self‐assessment
GDT	‐	0.450	0.652	0.429	<0.001	<0.001
IGDS9‐SF	−0.068	‐	0.752	0.930	<0.001	<0.001
GDSS	−0.042	0.027	‐	0.719	<0.001	<0.001
GAMES test	−0.076	−0.008	−0.035	‐	<0.001	<0.001
THL1	0.645	0.714	0.687	0.722	‐	0.160
Self‐assessment	0.490	0.559	0.532	0.567	−0.155	‐

*Note*: The values of the difference in Strength between IGD/GD operationalizations are below the diagonal, while the corresponding *p‐values* are above the diagonal.

### 3.1 Additional exploratory analysis

For descriptive purposes, we also outlined the profile of the negative outcomes of participants who scored above the respective thresholds in the IGD/GD measures. Detailed results are available at https://osf.io/8mykf.

## 4 DISCUSSION

Two important findings warrant further discussion: (1) despite ontological discrepancies between ICD‐11 and DSM‐5, IGD and GD constructs exhibited similar behavior within the networks of additional clinical features; and (2) compared to multiple item measures, both single self‐assessment items demonstrated significantly weaker associations with the additional clinical features within the networks.

(1) Regardless of the underlying ontology or specific operationalization, both ICD‐11 and DSM‐5‐based GD demonstrated the strongest correlations with all the additional clinical features (i.e., functional impairment). Even though a recent network analysis study (Adamkovič et al., 2023) reports significant alterations in IGD/GD symptoms network structure caused by minor differences in the symptom‐level operationalizations, it appears that, when GD is treated as a single score (instead of a complex mutually interacting system) and contains the pivotal symptoms as stated in the ICD‐11 (i.e., loss of control, preoccupation, and continued use), the effect of specific symptom operationalization is likely negligible. When modeling four IGD/GD scales (GAS7, IGDT10, GDT, and THL1) within one network, Billieux and Fournier (2022) found a strong item‐level construct overlap between the scales. This, however, does not eliminate the possibility that the scales could identify distinct populations (Karhulahti et al., 2022; Ko et al., 2020a), potentially due to differing scoring methods (monothetic vs. polythetic cut‐offs, different thresholds, etc.). The DSM‐5 or ICD‐11‐based IGD/GD central role in the networks does not appear to be affected by the addition of the additional diagnostic features. Had the DSM‐5, compared to the ICD‐11, included non‐relevant symptoms (e.g., as assessed by the experts in the Delphi study; Castro‐Calvo et al., 2021), the psychometric meaning of the sum score would become unclear as it would contain a higher proportion of error (or construct‐irrelevant) variance. Subsequently, this could substantially reduce the centrality of GD within the respective networks of GD and its additional diagnostic features. If true, this warrants the possibility to synthesize results from studies that used instruments coming from both DSM‐5 and ICD‐11 ontologies, but only if the goal is to evaluate IGD/GD relationships to other phenomena, not the relationships between the symptoms themselves. Moreover, our findings provide support for the realist ontology and clinical relevance of GD (Rumpf et al., 2018) by documenting the strongest role of GD in networks involving functional impairments commonly experienced by some gamers.
(2) Interestingly, when disordered gaming was measured via a single self‐assessment item, its centrality dropped significantly. In individuals with GD, self‐assessment requires admitting gaming‐related problems which would warrant a clinical intervention. The personal aspect (i.e., I am the person who has a problem) threatens the already disrupted self‐esteem (Lemenager et al., 2020). This could prove difficult as players with GD often use gaming and their avatar as a substitute to the non‐game reality (Beard et al., 2017). This coping mechanism (Kardefelt‐Winther, 2016) seems to be further supported by a recent report from UK GD treatment centers (Sharman et al., 2022), in which only 13.3% of treatment‐seekers were self‐referred.

### 4.1 Limitation

One of the two operationalizations of GD self‐assessment was specifically developed for this study. As such, evidence of its validity has not been established yet. That said, this operationalization showed high correlations (*r* from 0.47 to 0.62) with other operationalizations of IGD/GD and performed similarly to THL1 in the network.

## 5 CONCLUSION AND FUTURE DIRECTIONS

In this exploratory study, we investigated how different operationalization of IGD and GD relates to IGD/GD functional impairment suggested in the diagnostic manuals. Irrespective of the ontology or operationalization, both DSM‐5 and ICD‐11‐based measures showed the highest centrality within the network of GD and its additional clinical features. However, the strength centrality dropped when problematic gaming was assessed via single‐item self‐assessment measure, emphasizing the importance of using more complex GD measures. Future research on GD and its causes, or adverse consequences, is, however, needed to (dis)confirm these findings. Due to the lack of studies examining associations of GD operationalized according to ICD‐11 with other variables, the recent meta‐analysis (Ropovik et al., 2022) does not report a moderation analysis examining the effect of the ontology (DSM‐5 vs. ICD‐11) on the findings. In future meta‐analyses, it could be beneficial to compare whether the heterogeneity in the observed effect sizes can be attributed to the different underlying ontology. Future research could also explore diagnostic accuracy and utility of single self‐assessment items.

## AUTHOR CONTRIBUTIONS

**Marcel Martončik**: Conceptualization; methodology; investigation; data curation; resources; writing ‐ original draft; writing ‐ review & editing. **Matúš Adamkovič**: Conceptualization; methodology; formal analysis; data curation; writing ‐ original draft; writing ‐ review & editing. **Ivan Ropovik**: Conceptualization; methodology; writing ‐ review & editing.

## CONFLICT OF INTEREST STATEMENT

The authors declare no conflicts of interest.

## ETHICS STATEMENT

Permission to conduct the study was approved by the Ethics committee of the Faculty of Arts University of Presov.

## Data Availability

All data, R script, and materials related to this study are available on the OSF repository: https://osf.io/6a7kv/.

## ENDNOTES

^1^
Namely: (1) preoccupation with Internet games, (2) withdrawal symptoms, (3) increased tolerance, (4) loss of control over gaming, (5) loss of interests in other hobbies, (6) continued use despite problems, (7) deception regarding the amount of gaming, (8) escape motive, (9) jeopardizing or losing significant relationships or opportunities.

^2^
Note: EBICglasso was chosen as the primary estimation method due to its proven performance under conditions similar to those anticipated for this study (see Isvoranu & Epskamp, 2023). The tuning parameter of 0.25 was selected to achieve a balance between density and sparsity of the estimated networks.
